# Axonal blockage with microscopic magnetic stimulation

**DOI:** 10.1038/s41598-020-74891-3

**Published:** 2020-10-22

**Authors:** Jordan Skach, Catherine Conway, Lauryn Barrett, Hui Ye

**Affiliations:** 1 Department of Biology, Loyola University Chicago, Chicago, IL USA; 2 Department of Biology, Quinlan Life Sciences Education and Research Center, Loyola University Chicago, 1032 W. Sheridan Rd., Chicago, IL 60660 USA

**Keywords:** Computational biophysics, Biomedical engineering, Neurology, Neurological disorders, Neuropathic pain, Computational models, Computational neuroscience

## Abstract

Numerous neurological dysfunctions are characterized by undesirable nerve activity. By providing reversible nerve blockage, electric stimulation with an implanted electrode holds promise in the treatment of these conditions. However, there are several limitations to its application, including poor bio-compatibility and decreased efficacy during chronic implantation. A magnetic coil of miniature size can mitigate some of these problems, by coating it with biocompatible material for chronic implantation. However, it is unknown if miniature coils could be effective in axonal blockage and, if so, what the underlying mechanisms are. Here we demonstrate that a submillimeter magnetic coil can reversibly block action potentials in the unmyelinated axons from the marine mollusk *Aplysia californica*. Using a multi-compartment model of the *Aplysia* axon, we demonstrate that the miniature coil causes a significant local depolarization in the axon, alters activation dynamics of the sodium channels, and prevents the traveling of the invading action potentials. With improved biocompatibility and capability of emitting high-frequency stimuli, micro coils provide an interesting alternative for electric blockage of axonal conductance in clinical settings.

## Introduction

Since numerous neurological dysfunctions are characterized by undesirable nerve activity, reversible nerve blockage holds promise in the treatment of these conditions. Drugs are commonly used, but their effects are slow and sometimes contain side effects such as systemic toxicity^1^. Axonal conductance can be controlled with electrodes positioned close to the axons, namely, extracellular stimulation. High-frequency stimulation (HFS) with an electric current at several hundred to several thousand hertz, has proved to be a reliable method for suppressing axonal conduction since it produced a quick and reversible focal block at the site of the stimulating electrode^2–4^. Nerve conduction blockage with such stimulation has been confirmed in various biological preparations, including pudendal nerve^5^, sciatic nerve, vagus nerves^6^, afferent axons in the hippocampus^7,8^, and cardiac tissue^9^.

Clinically, HFS on the pudendal nerve was implemented for the treatment of the detrusor-sphincter dyssynergia, since it can block sphincter contractions and leads to its relaxation^10^. HFS was also used for restoring function in the upper extremity, lower extremity, and respiratory system^11^. HFS blockage of axonal conductance also provided an interesting control diagram of deep brain stimulation (DBS) of the subthalamic nucleus (STN), for the treatment of Parkinson’s disease. A possible mechanism of DBS is that it causes axonal and synaptic failure in the STN projections, decoupling STN from its synaptic targets, basal ganglia output nuclei^8,12^.

Despite the clinical potential of implanted electrodes for HFS axonal blockage, some of its side effects can not be completely avoided. For example, lead migration and electrode fracture could happen after chronic implantation^13^. Therapeutic effects can be largely altered by the inflammatory and immune responses due to the direct contact between the stimulating electrode and the tissue^14,15^. The formation of glial scarring around the individual electrode^16,17^ can block electric currents delivered by the electrode, resulting in unpredictable changes or loss of neural response. Therefore, the biocompatibility of the implanted electrodes could be a major concern in implanted device design^16^.

An alternative method to generate an electric current inside the neural tissue is via electromagnetic induction by magnetic coils. The induced electric fields in the neural tissue stimulate the neurons^18,19^. Recently, miniature-sized magnetic coils have been used to activate selected neuronal subpopulations^20,21^. If implanted under the cover of insulated biocompatible materials, miniature coils offer several advantages in biocompatibility and operational feasibility^22^. They prevent the direct contact between the electrode and neural tissue, eliminating numerous problems that may arise at the brain-electrode interface^16,23,24^. Theoretically, these practices can increase stimulation focality on the target tissue and avoid the adverse effects associated with implants^25–27^. Despite the obvious advantage of using a miniature magnetic coil in axonal blockage, this possibility has never been experimentally proven and mechanistically studied.

The marine mollusk, *Aplysia californica*, provides an appealing system for the study of axonal blockage by the miniature coil. Previous studies have used the *Aplysia* nerves to study the blockage of unmyelinated axons with Kilohertz high-frequency alternating current^28,29^, or with infrared^29,30^. Hereby, we will study the magnetic blockage of axonal conductance using the neurons in the buccal ganglion and their associated buccal nerves in *Aplysi*a. These nerves carry unmyelinated axons of several important motor neurons and interneurons. The locations of these in the buccal ganglion have been previously identified^31–37^. Neural activity in these neurons is responsible for the contraction of specific muscles and the generation of feeding responses in the live animals^38,39^. Furthermore, a detailed multi-component NEURON model of the buccal neuron has been built for the study of the neuronal response to electric stimulation^31^.

To explore whether a miniature coil could indeed block axonal conductance at a high frequency band, we used a combined approach of electrophysiological experiments and computational modeling. We designed a system that can deliver sufficient electric current of various frequency and intensity into a commercially available miniature coil. The size of the miniature coil is 1 mm × 0.5 mm × 0.5 mm, matching the size of the clinical DBS electrode and tungsten electrode for animal brain implantation. We recorded axonal conductance in the buccal nerve II (BN2) of the buccal ganglion in *Aplysia californica* while applying magnetic stimulation to the axon. To understand the mechanisms of axonal blockage with the miniature coil, we calculated the magnetically-induced electric field inside the axon. We then applied this induced electric field to a multi-compartment model of *Aplysia* axon using NEURON simulation and studied the ionic channel dynamics underlying the coil-induced axonal blockage.

## Results

### Axonal blockage by high frequency stimulation with a miniature coil

**Figure 1:**
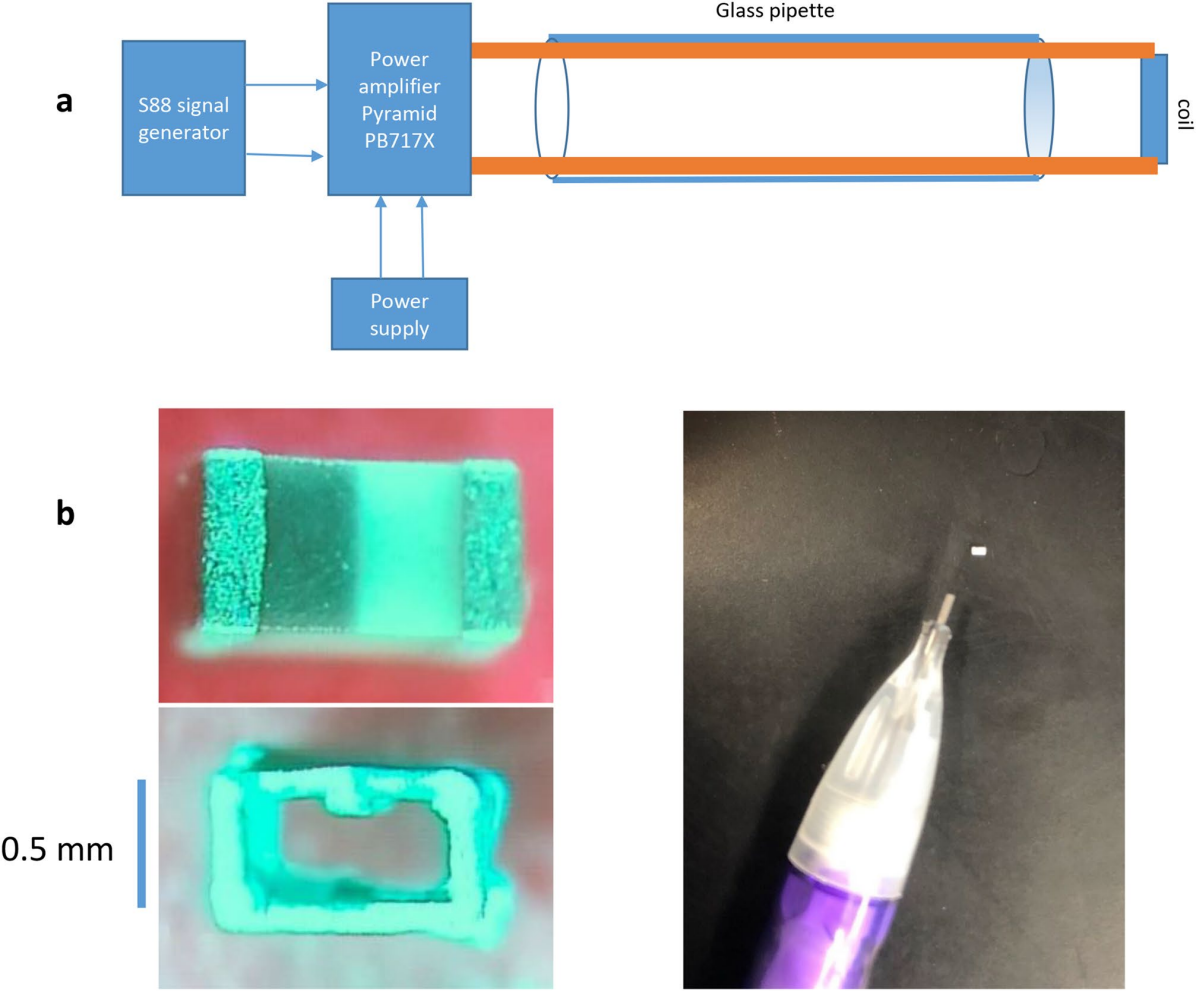
Magnetic field generator. (**a**) The generator included a signal generator, a power amplifier, and a coil held by a glass tube. (**b**) Image of the miniature coil used for the electrophysiology experiment. The ceramic cover of the inductor was chemically dissolved to expose the structure of the inside solenoid. Size of the coil was compared with the tip of a mechanic pencil.

We designed a system that could deliver electric currents to the miniature coil with controllable frequency and intensity. The magnetic generator included a signal generator, a power amplifier and the miniature coil (Fig. 1a). A commercial multilayer surface mount inductor was selected for the study due to its small size and capability of producing a large electric field. To further reveal the internal structure of the coil, we chemically dissolved the coil encapsulation (Fig. 1b). Each inductor contained 20 loops of rectangular shape (1 mm × 0.5 mm). Using the buccal ganglion and its associated BN2 from the marine mollusk *Aplysia californica,* we performed a series of electrophysiological experiments to investigate the capability of a miniature coil in axonal blockage.

#### HFS with miniature coil suppressed action potentials in an isolated nerve

**Figure 2:**
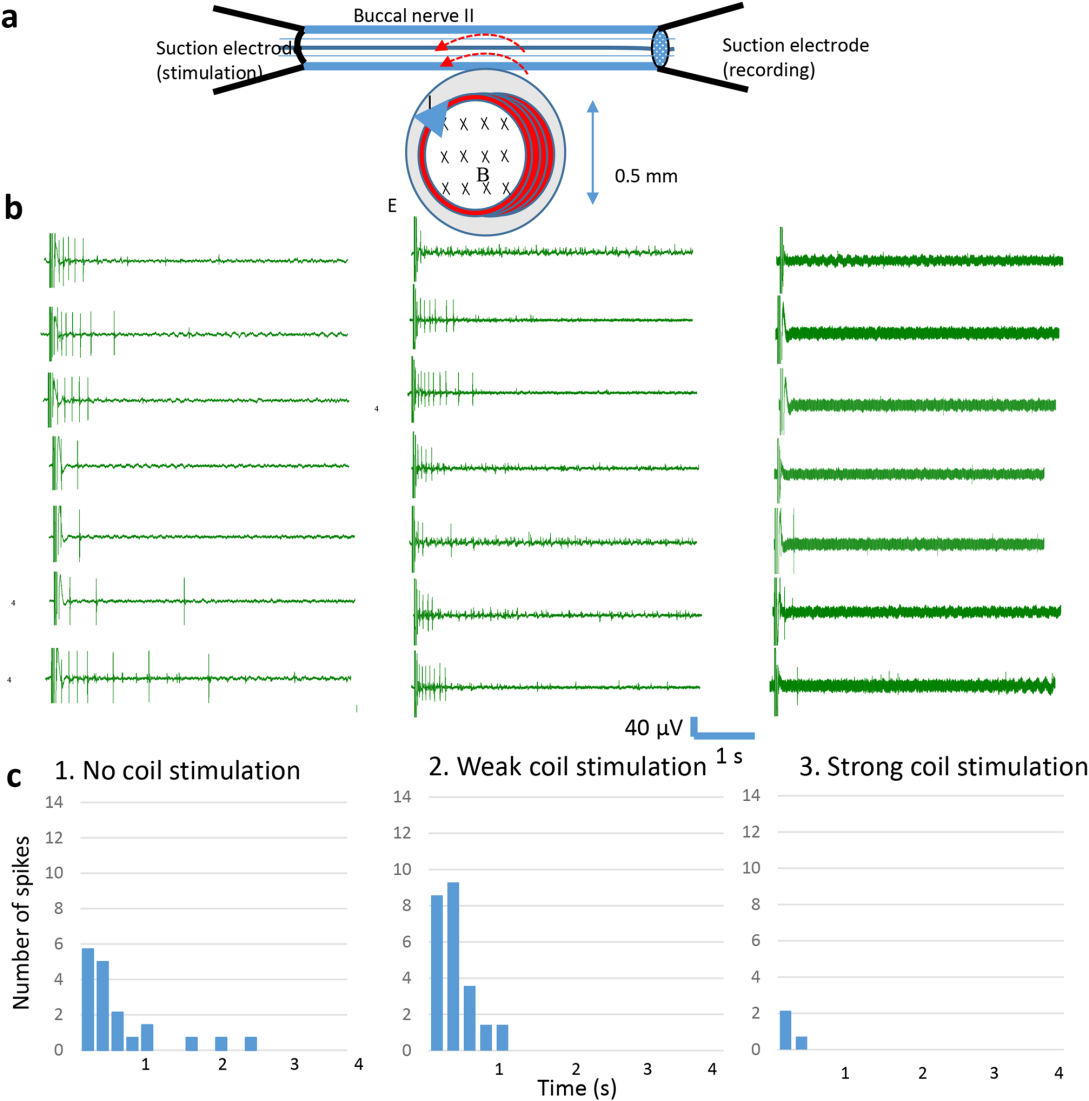
Miniature coil suppressed axonal conductance in the isolated buccal nerve II (BN2). (**a**) Experimental setup for conductance blockage in an isolated BN2 with the miniature coil. The miniature coil was positioned next to the BN2 to induce an electric field parallel to the axon. (**b**) Action potentials were generated by stimulating one end of the BN2 with electric pulses. Response recorded from the other end of the nerve by a suction electrode consisted of a short stimulus followed by a series of action potentials. 1. Axonal conductance when no magnetic stimulation was applied to the BN2. 2. Axonal conductance when a low intensity magnetic stimulation was applied. 3. Axonal response when a high intensity magnetic stimulation was applied. C. Histogram of the firing frequency of the seven repetitions shown in (**b**).

To elicit action potentials in the isolated BN2, we delivered electric pulses (0.1 ms duration, 4 s interval) to one end of BN2 via a suction electrode. We recorded action potentials on the distal end with another suction electrode (Fig. 2a). To elicit activities from a minimal number of axons in the BN2, we gradually decreased the stimulation intensity to a level that only a few action potentials with similar sizes were triggered, meaning a minimal number of axons were activated. There were slight variations in the number and latency in the elicited spikes from trial to trial, but generally, a relatively higher frequency of action potentials were elicited right after the electric pulses and the frequency decreased 1–2 s after the pulses (Fig. 2b).

To investigate the threshold of magnetic stimulation for axonal blockage, we applied 400 Hz square signal to the miniature coil via the power amplifier with two intensities, a weak signal (1 V) and a strong signal (10 V). We found that strong stimulation was capable of suppressing axonal action potentials while the weak stimulus could not (Fig. 2b). To quantify the effects of the magnetic field in axonal blockage across each stimulation intensity, we averaged the frequency of the spikes from multiple trials and plotted their distributions as histograms (Fig. 2c). Similar responses were seen in five isolated BN2 in which strong magnetic stimulation suppressed action potentials in BN2, and weak stimulus did not.

#### HFS with miniature coil suppressed action potentials generated by antidromic stimulation

**Figure 3:**
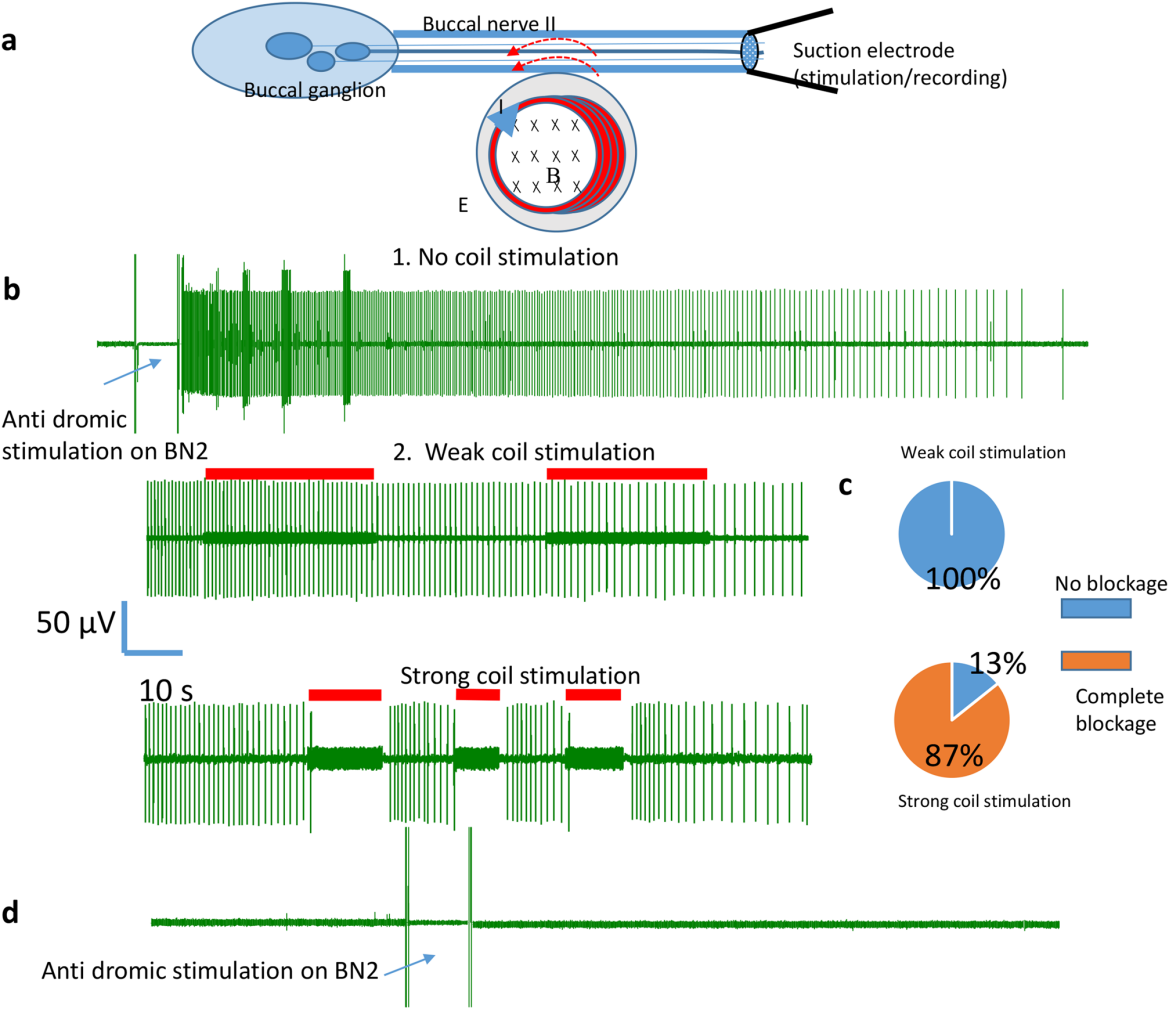
Miniature coil suppressed action potentials generated by antidromic stimulation of the buccal ganglion. (**a**) Experimental setup for antidromic stimulation of soma in the buccal ganglion. A suction electrode was applied to the distal end of BN2. High frequency pulses were applied to the nerve via the suction electrode to elicit action potential in the buccal ganglion. The same electrode then recorded action potentials generated by soma in the ganglion. The miniature coil was positioned next to the BN2 to induce an electric field parallel to the axon. (**b**) 1. Antidromic stimulation elicited burst of activities in the BN2, which usually contained several different size groups and lasted for approximately 2 min. Then the firing frequency gradually decreased until the nerve became quiescent. 2. Weak magnetic stimulation did not block the activities in BN2. 3. Strong magnetic stimulation completely suppressed axonal conductance. Red bars represented the magnetic stimulation. (**c**) Percentage of complete blockage in BN2 activity by the magnetic field of weak or strong intensities in multiple trails. D. BN2 was transected close to the buccal ganglion. The suction electrode on the BN2 could not record any nerve pulses after the same antidromic stimulation protocol was applied.

Large cell bodies of identified neurons B3, B6, B9, and B10 in the buccal ganglion send axons to the BN2^40^. The firing of these neurons generates large spikes in the extracellular recordings from the BN2^32,38^. Antidromic stimulation of the BN2 activates these neurons in the buccal ganglion. In this set of experiments, a suction electrode was applied to the distal end of the BN2 for stimulation and recording purposes (Fig. 3a). When a 20 Hz pulse train was delivered to the BN2 for several seconds, it elicited a burst of activities in the BN2. These spikes lasted for 1–3 min, then slowed down and disappeared. The spikes were of several different sizes, indicating several motor neurons were activated (Fig. 3b). Under this stimulation protocol, the 2nd largest units were most prominent, likely due to the activation of B6 neuron^32^.

Weak and strong magnetic fields (400 Hz) were used to block these action potentials (Fig. 3b). Under weak stimulation, axonal conductance was suppressed in the 1 out of 7 trials (13%, Fig. 3c). Under strong stimulation, axonal blockage was observed in 12 out of 12 trials (100%). This difference is statistically significant $({x}^{2}=11.328, p<0.001).$ The only time that action potential was blocked by the weak magnetic field was when the frequency of BN2 activity was extremely low (< 1.4 Hz). Similar data was observed in five buccal ganglion preparations. To confirm that the activities recorded in distal BN2 were indeed generated by the cell bodies in the buccal ganglion, we transected the proximal end of the BN2 close to the buccal ganglion. This transection permanently eliminated the BN2 spikes (Fig. 3d).

#### HFS with miniature coil suppressed action potentials generated by specific soma activation

**Figure 4:**
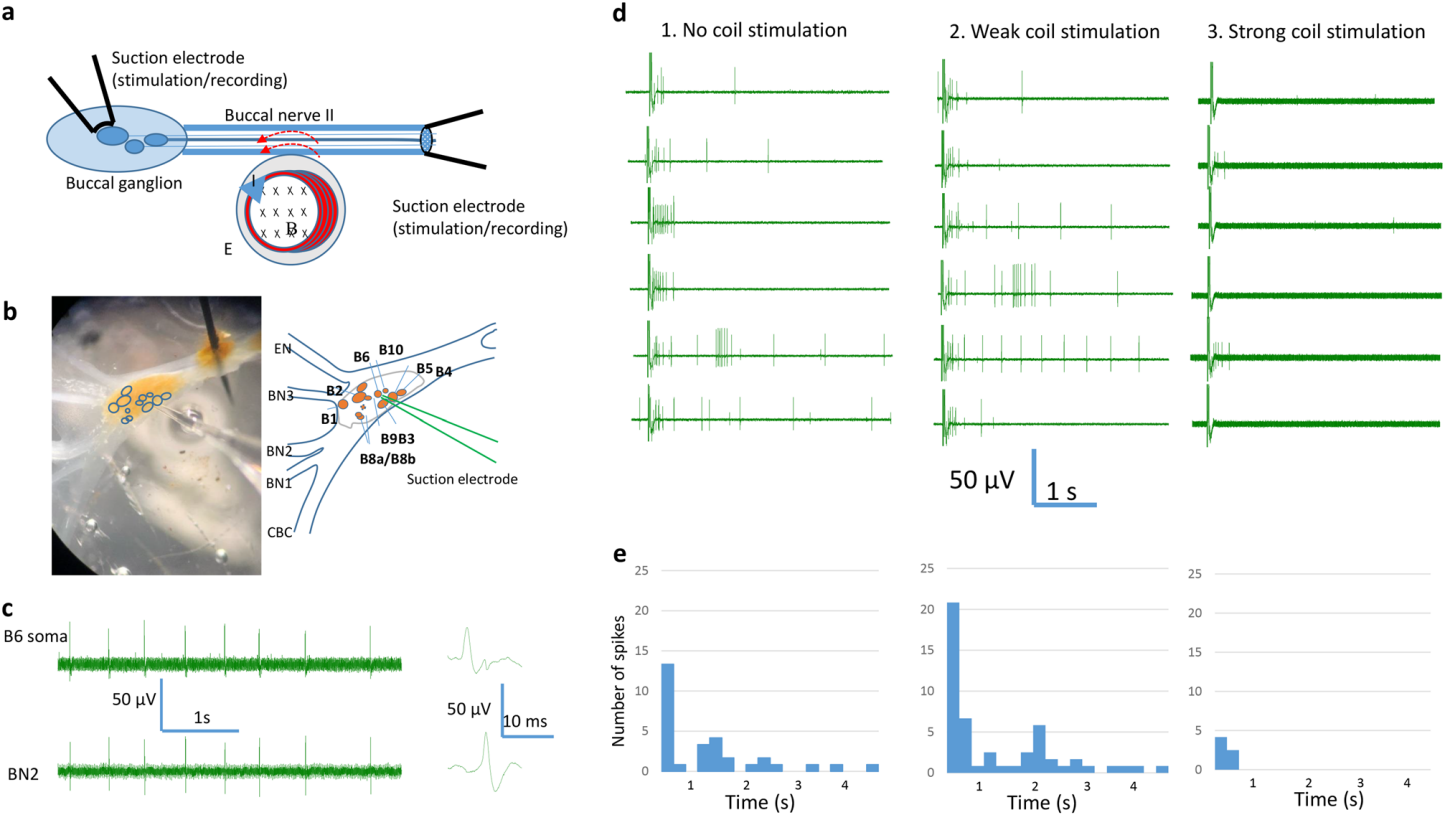
Miniature coil suppressed action potentials generated by extracellular B6 soma stimulation. (**a**) The miniature coil was positioned next to the BN2 to induce an electric field parallel to the axon. Soma activity was initiated by the extracellular electrode positioned on the B6 soma in a partially desheathed buccal ganglion. (**b**) Morphological location of the B6 neuron on the caudal surface of the buccal ganglion. Left picture showed the buccal ganglion. The right schematic was drawn based on the left picture. The picture and schematic together indicated the locations of several identified interneuron (B4 and B5), motor neurons whose axons innervates BN2 (B3, B6, B9, B10), and some other neurons. The sketch also showed the buccal nerves. A suction electrode was applied to the partially desheathed ganglia membrane, right above the B6 neuron. (**c**) Electrophysiological identification of the B6 neuron. Extracellular recording of spontaneous B6 soma activity showed a one-to-one relationship with the axon activity. (**d**) Action potentials were triggered in the B6 soma with electric pulses (0.1 ms width, 4 s interval). 1. Axonal conductance when no magnetic stimulation was applied to the BN2. 2. Axonal conductance when a weak magnetic stimulation was applied to BN2. 3. Axonal response when a strong magnetic stimulation was applied to BN2. Note the thick baseline that represented the magnetic induced noise. (**e**) Histogram of the firing frequency of the six repetitions shown in (**d**).

Results presented so far suggest that a population of axons has been inhibited by the miniature coil. Specifically, the 2nd largest units in BN2 were reliably inhibited by the magnetic field. Previous studies suggest that these units are generated by the B6 soma in the buccal ganglion^32^. To further test the hypothesis that high-frequency stimulation with a miniature coil could inhibit axonal activity from a single neuron, we identified the B6 neuron in the buccal ganglion using its morphological location. We then positioned a glass electrode on top of the soma to initiate action potentials in this neuron (Fig. 4a,b). We also applied a suction electrode on the BN2 to record axonal action potential. We observed a one-to-one relationship between the soma activity and BN2 activity, with the soma activity leading the axon activity by approximately 6 ms (Fig. 4c).

To better control trigger single neuron activity from B6, we delivered short current pulses to the soma electrode. The intensity of the pulses was adjusted so that several action potentials were triggered by a single electric pulse^32^. The extracellular electrode on the BN2 recorded some high frequency action potentials right after the electric pulses, and the frequency decreased 1–2 s after the pulses (Fig. 4d). In this experiment, because the extracellular amplifier was switched on stimulation mode, activity in the soma cannot be recorded from the soma electrode^32^.

When a stable response of the neuron was established, we delivered 400 Hz square waves to the coil with low and high intensities. The weak stimulation failed to block the axon. Strong stimulation with 400 Hz square waves significantly suppressed axonal conductance (Fig. 4d). To quantify the effects of the magnetic field in axonal blockage, we averaged the frequency of the spikes recorded under specific magnetic stimulation from multiple trials and plotted their distributions as histograms (Fig. 4e). Similar responses were seen in three preparation in which strong magnetic stimulation blocked axonal action potentials that were generated by B6 soma stimulation.

#### Axonal suppression by the miniature coil was local to the stimulation site

**Figure 5:**
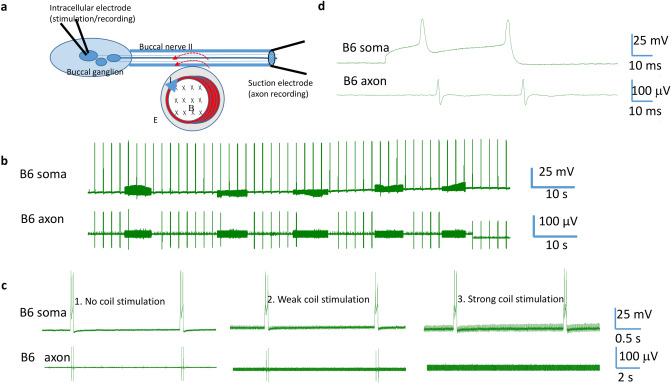
Miniature coil suppressed action potentials generated by intracellular B6 soma stimulation. (**a**) Experimental setup of the miniature coil for axon stimulation. The miniature coil was positioned next to the BN2 to induce electric field parallel to the axon. An intercellular electrode was inserted to the B6 neuron to trigger and record action potentials. The distal axon end of the B6 neuron was recorded by a suction electrode on the BN2. (**b**) One-to-one relationship was observed in the soma and in the axon of B6 neuron when the soma was activated by a short depolarization pulse. (**c**) Strong coil stimulation reversibly block axonal conductance. (**d**) Effects of stimulation amplitude in axonal suppression. 1. Axonal conductance when no magnetic stimulation was applied to the BN2. 2. Axonal conductance when a weak magnetic stimulation was applied to BN2. 3. Axonal conductance when a strong magnetic stimulation was applied to BN2, which blocked the action potential traveling.

Controlling single neuron activity using extracellular stimulate does not require desheathing of the buccal ganglion. However, precise control in the timing of the neuron activity is difficult with extracellular stimulation since the electrode is positioned outside the cell. One cannot observe soma activity when the extracellular electrode was used for stimulation^31,32^. One could suspect that the absence of the action potentials in the axon during coil stimulation could be due to a direct inhibitory effect on the soma rather than the axon. To solve these puzzles, we desheathed the buccal ganglion and impaled the B6 soma with an intracellular electrode (Fig. 5a). We observed action potential propagation from the soma to the distal axon end in a few milliseconds (Fig. 5b). To have precise control of soma activity, we delivered 1 Hz pulses to the soma. We then applied strong magnetic stimulation to the axon and observed a quick blockage of axonal conductance, while the soma was not affected (n = 4). Axonal blockage was rapidly reversible (Fig. 5c) when the magnetic field was withdrawn. Weak coil stimulation, however, did not block axonal conductance in the B6 neuron (Fig. 5d).

### Computation of miniature-coil induced electric field and activating function

**Figure 6:**
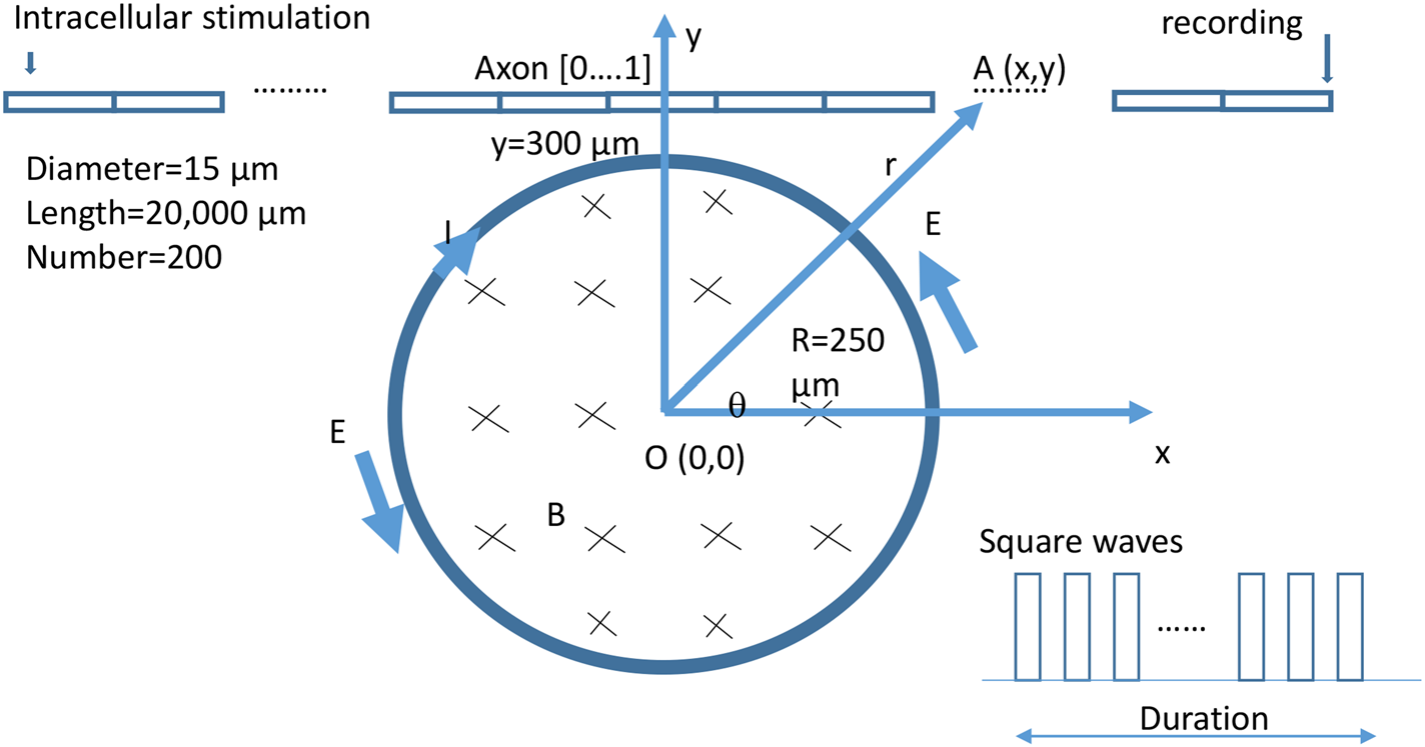
NEURON modeling of an unmyelinated axon under magnetic stimulation. The multi-compartment H/H type of modeled axon was 20,000 µm in length and was divided into 200 segments. Each segment was a cylinder of length 100 µm and diameter 15 µm. An intracellular electrode was used to initiate action potentials on the left end of the axon, and another electrode recorded the arriving action potential on the right end of the axon. Distance between a point A(x,y) on the axon and the center of the coil point O (0,0) was r. Coil radius was R. Electric pulses of high frequency were delivered into the coil and generated an induced electric field (E) in the counterclockwise direction.

**Figure 7:**
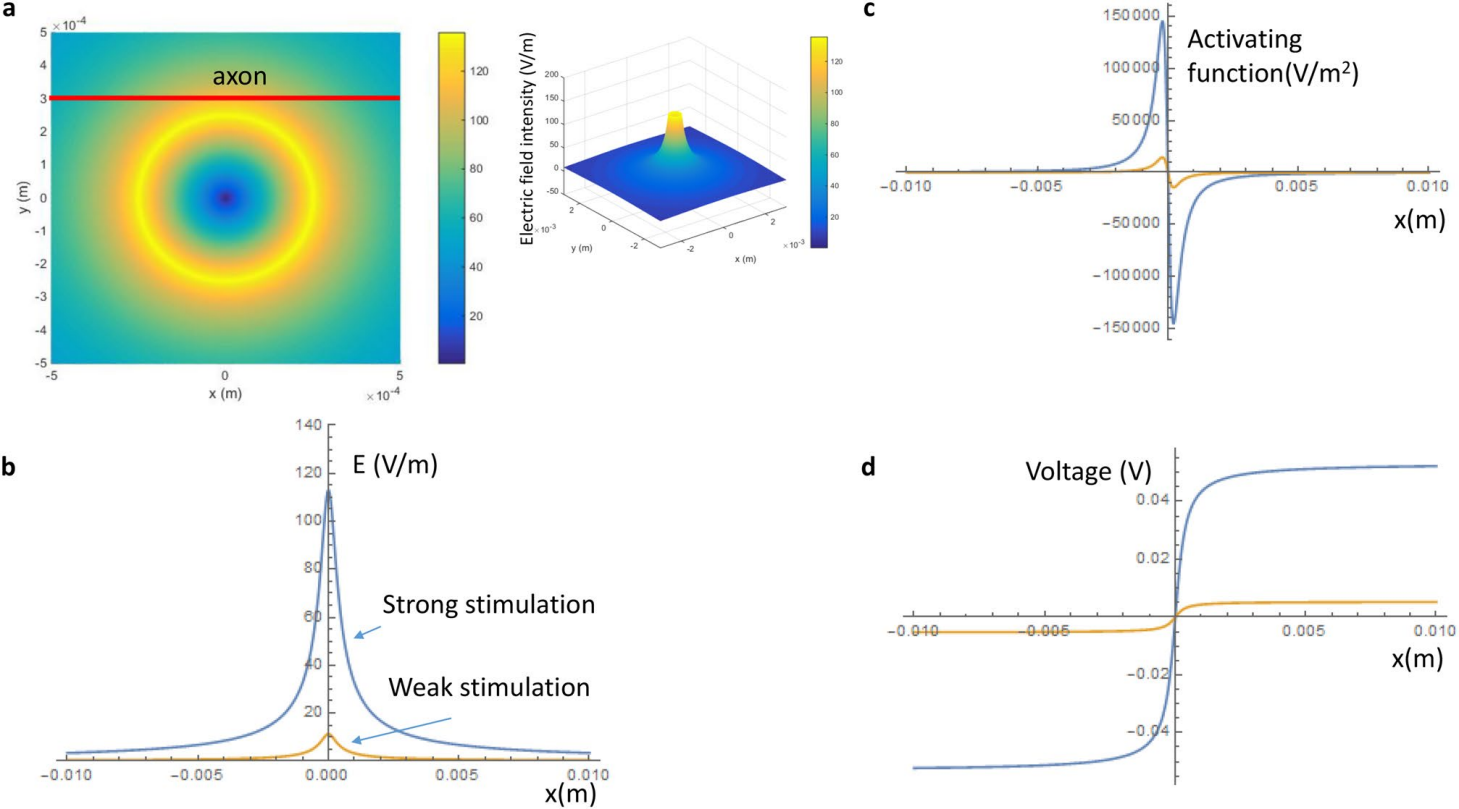
Miniature coil induced electric potential and its gradient along the axon. (**a**) Distribution of the induced electric intensity around the coil. The red line represents the location of the axon. (**b**) Induced electric field along the axon. (**c**) Activating function (dE/dx) along the axon. (**d**) Induced voltage along the axon. Blue curves: strong stimulus; Yellow curves: weak stimulation.

Previous works suggest that level of neural activation was determined by the intensity of the electric fields^41–43^. We used biophysics modeling to estimate the intensities of the induced electric fields inside the axons for our electrophysiology experiments. For simplicity, we modeled the coil as a circular cylinder with radius 250 µm and infinite length (Fig. 6). Taking into account the coated layer of the miniature coil (approximately 50 µm in thickness), we estimated that the center of the coil was approximately 300 µm away from the axon. Figure 7a shows the distribution of the induced electric field around the coil. The induced electric field decayed quickly as a function of 1/r, where r is the distance between the center of the coil to its target (Eq. (8)).

For the “weak stimulation”, which was incapable of axonal blockage, the 1 V square wave signal generated a 0.2 V voltage drop across the coil leads. Based on Eq. (8), this produced an approximately 10 V/m electric field inside the axon. For the “strong stimulation”, which blocked axonal conductance, the 10 V square signal generated a 2.16 V square voltage across the coil leads. This produced an electric field as large as 110 V/m (Fig. 7b).

The activating function is defined as the gradients of the electric field along the axon^44^. It predicts the location and speed of depolarization or hyperpolarization by the extracellular stimulation^45^. Figure 7c demonstrates the activating function along the axon, which contains a depolarization zone and a hyperpolarization zone. For the “weak stimulation”, the induced electric field produced a maximal field gradient of 14,000 V/m^2^. For the “strong stimulation” that could suppress axonal conductance, the maximal activation function was 140,000 V/m^2^, well above the value that can cause neural activation (50,000 V/m^2^ in^25^).

### Cellular and ionic mechanisms of axonal blockage by the magnetic stimulation

**Figure 8:**
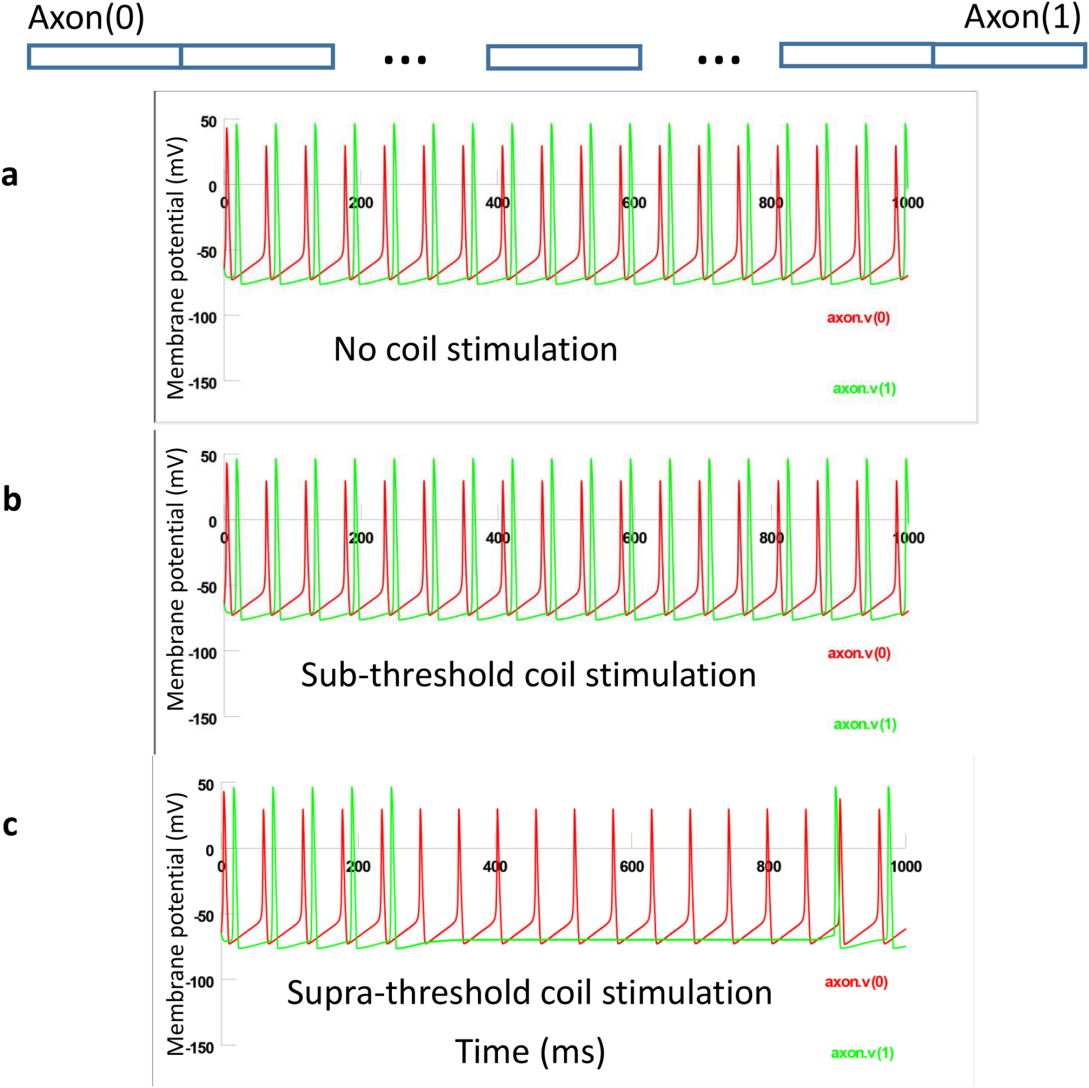
Axonal blockage by the miniature coil and it dependency on stimulus intensity in a NEURON model. The miniature coil was positioned close to the mid-point of the 20,000 µm long axon, 300 µm away from the axon. Duration of coil stimulation was 500 ms. Stimulation frequency was 400 Hz square pulses. (**a**) Physiological action potentials were triggered by intracellular injection of 10 nA current and they travelled from left (axon(0)) to the right side (axon(1)). (**b**) Weak, subthreshold coil stimulation did not affect axonal conductance. (**c**) Strong, supra threshold coil stimulation completely suppressed the traveling of action potentials.

NEURON modeling was used to investigate the underlying mechanisms of axonal blockage by the high frequency field generated by the miniature coil. In a typical stimulation protocol, action potentials were initiated in the left end of the axon (axon(0)), by an intracellular electrode. These action potentials propagated along the axon to its right end (axon(1), Fig. 8a, Supplementary Video 1). For stimulation, the coil was located close to the mid-point of the axon (axon(0.5), Fig. 6). We applied 500 ms, 400 Hz magnetic stimulation to the axon. The magnitude of the extracellular voltage (Fig. 7d) was calculated with Eq. (11) and was applied to the NEURON model. Under weak, subthreshold stimulation, axonal conductance was not affected (Fig. 8b, Supplementary Video 2). Under strong, supra threshold stimulation, axonal conductance was completely blocked (Fig. 8c, Supplementary Video 3).

**Figure 9:**
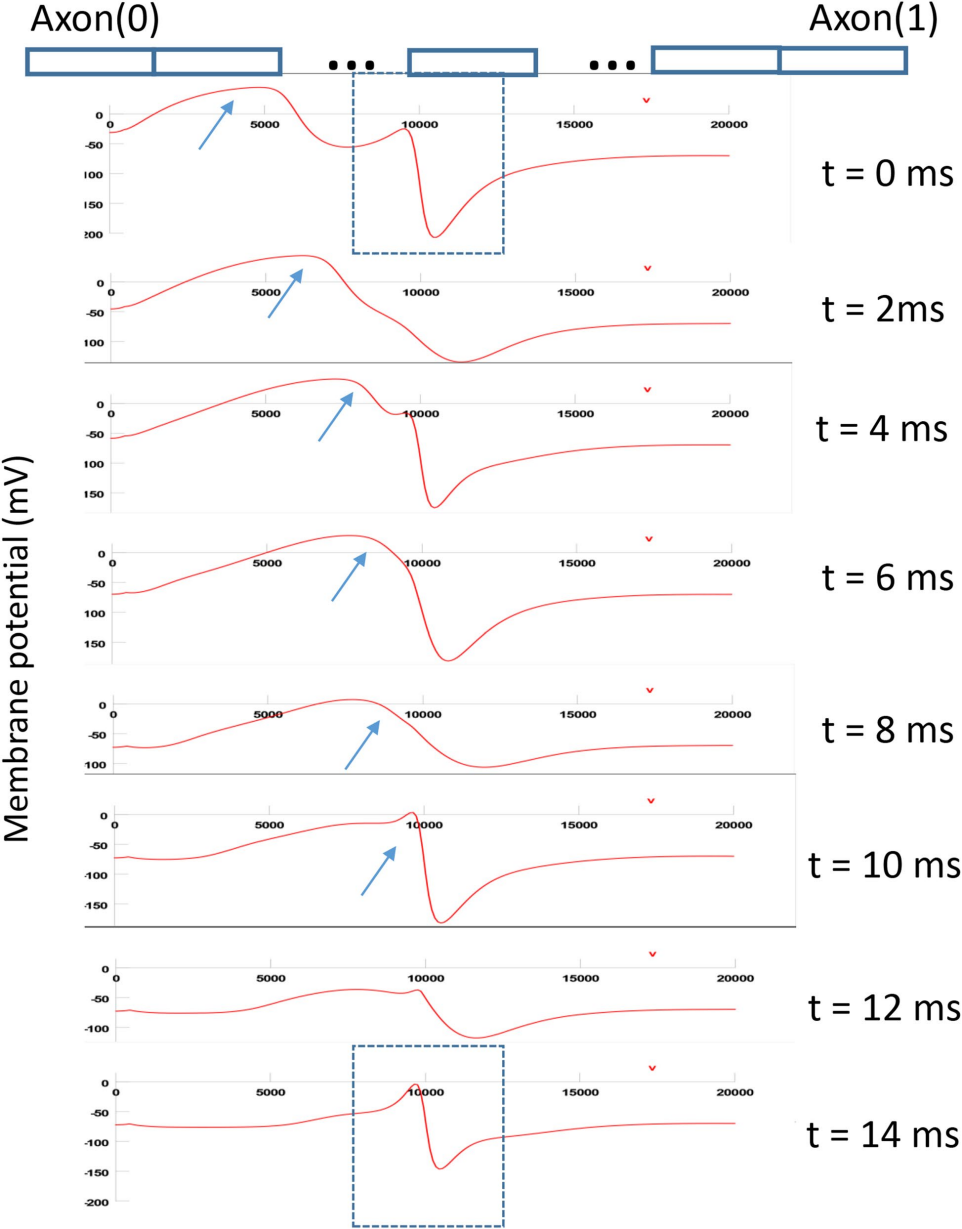
Space plot of membrane voltage changes during coil stimulation. Arrows indicated the invading action potential. The dash-lined blue square represented the location of the coil-induced depolarization/hyperpolarization area.

To visualize the propagation of action potential and its blockage by the coil, we generated a space plot of the membrane voltage along the axon (Fig. 9). In this example, one single action potential traveled along the axon. At t = 0 ms, the action potential depolarized the axonal and propagated to the right side of the axon. The coil produced a depolarization zone and a hyperpolarization zone in the middle of the modeled axon, as predicted by the activating function analysis (Fig. 7c). For the 400 Hz square signal, this depolarization/hyperpolarization zone lasted for 1.25 ms during the “turn on” period of the coil. At t = 4 ms, the invading action potential approached the depolarization zone. At t = 6 ms, the action potential invaded the depolarization zone, collided and merged with the coil-induced depolarization. From t = 8 to 14 ms, the invading action potential failed to pass the depolarization zone and faded before it reached the midpoint of the axon. The hyperpolarization zone of the axon was merely affected by the invading action potential. In summary, the coil generated a “depolarization zone” blocked the invading action potentials.

Since excessive membrane depolarization can cause inactivation of the voltage-dependent sodium channels^46^, we hypothesized that axonal segment in the depolarization zone lost its capability of initiating action potentials due to the inactivation of the sodium channels in this area.

**Figure 10:**
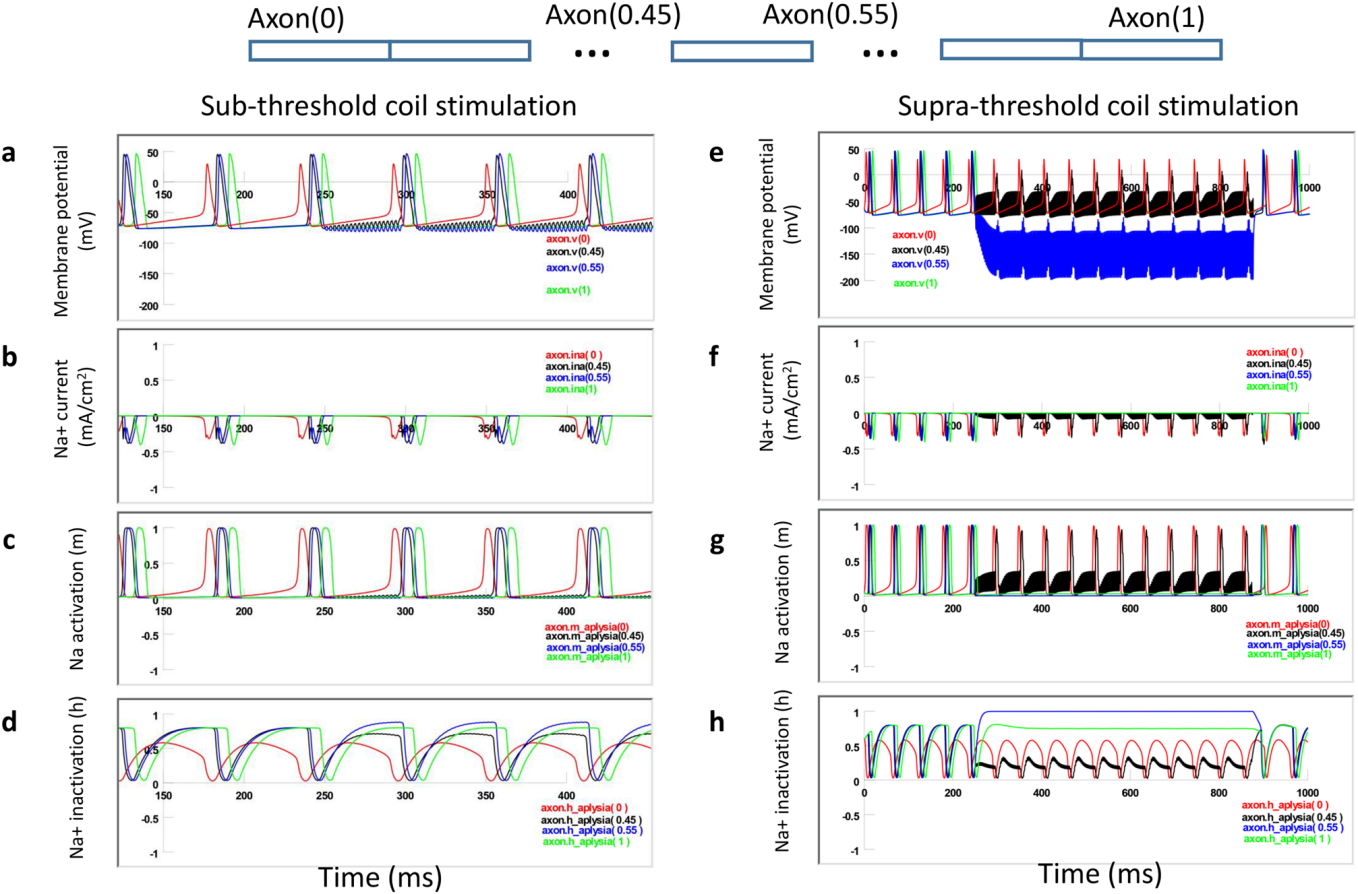
Mechanisms of axonal blockage by the miniature coil. (**a**–**d**) Weak, subthreshold coil stimulation. Membrane potential (**a**), Na^+^ current (**b**), sodium channel activation m (**c**) and sodium channel inactivation h (**d**) in several axonal segments during subthreshold stimulation. (**e**–**h**) Strong, supra-threshold stimulation that completely blocked the axonal conductance. Membrane potential (**e**), Na^+^ current (**f**), sodium channel activation m (**g**) and sodium channel inactivation h (**h**) in several axonal segments during supra-threshold stimulation. Axon(0): location of action potential initiation. Axon(0.45): depolarization zone. Axon(0.55): hyperpolarization zone. Axon(1): end of the axon.

To test this hypothesis, we plotted the inward sodium current (INa^+^), sodium activation (h) and inactivation (m) variables in the depolarization zone (Fig. 10, axon(0.45)). In the absence of coil stimulation, the membrane was at resting potential (− 65 mV). The sodium channel was sufficiently inactivated (h = 0.8). This allowed the invading action potential to fully activate the sodium channels (m = 1) to produce large inward sodium current (Ina), and the invading action potential could travel through the zone. Under weak, sub-threshold stimulation, sodium inactivation was slightly decreased (h = 0.6) at rest. The invading action potential could still sufficiently activate the sodium channel (m = 1) and traveled through this region (Fig. 10a–d).

Under supra-threshold stimulation, coil-induced membrane depolarization caused partial activation (m = 0.2) of sodium channels and insufficient inactivation (h = 0.2) at rest. Depolarization also reduced the driving force for sodium. As a consequence, the invading action potential couldn’t fully activate the sodium channel (m = 0.9) and generate significant inward INa^+^, preventing the initiation of a full action potential (Fig. 10e–h). Theoretically, the hyperpolarization zone could also provide another region for action blockage, since excessive hyperpolarization could cause failure of sodium channel activation^46^. In conclusion, coil stimulation caused local membrane depolarization, leading to the sodium channel inactivation and failure of sustaining the axonal action potentials.

## Discussion

A lack of research on the magnetic blockage of axonal conductance and the underlying mechanisms are likely due to several technical challenges. First, in order to block axonal conductance, focal stimulation is needed to modulate neural activity at a microscopic cellular level, and manufacturing coils with such small dimensions is challenging. Second, it is not known if the submillimeter coil can generate sufficient electric fields for axonal blockage, including the nature of the stimulus signal that can be effective in axonal suppression. Third, the practice of magnetic stimulation of neural tissue causes large noise, which is challenging for electrophysiology confirmation of axonal blockage. Finally, for mechanistic study with computer modeling, it is challenging to implement experimental parameters that includes both the coil parameters and biologically authentic axons.

In this paper, we solved these problems by using a commercially available miniature coil and applied extracellular and intracellular techniques to the buccal ganglion of *Aplysia californica.* Furthermore, we used biophysics modeling and NEURON simulation of an *Aplysia* axon for the quantitative understanding of axonal blockage by the magnetic stimulation. There are two major conclusions that can be made from the study. First, this paper is the first to demonstrate that by delivering high frequency pulses, the miniature coil can completely suppress axonal conductance in the unmyelinated axons. Axonal blockage was observed in a large number of axons (Fig. 3), a few axons (Fig. 2), or in a single axon (Figs. 4, 5) that is actively conducting action potentials.

Second, this paper is the first to demonstrate that axonal blockage is due to the extensive depolarization in the local axonal segments. Previously, we have shown that magnetically-induced currents could cause direct membrane polarization with a biophysics model of unmyelinated axon^47^. This work further revealed that axonal blockage was due to altered sodium channel dynamics. In HFS with an electrode, it was also found that depolarization could lead to the inactivation of sodium channels and impair the mechanisms of neuronal firing^48^. Therefore, HFS stimulations with coil or electrode share some similar mechanisms in axonal blockage.

### Miniature coil technique for axonal blockage

Results from this paper provide several insights for the further development of the miniature coil technology for the axonal blockage.

#### Importance of high frequency stimulation using miniature coil

The NEURON model suggests that maintaining a sufficient period of local depolarization is essential in blocking the invading action potential (Fig. 10). In the conventional method of neural stimulation using implanted electrodes, direct current (DC) can be delivered to the axon to achieve this goal. For magnetic stimulation, an electric field is generated via electromagnetic induction. Maintaining a constant induced electric field for a long period of time is challenging since this requires the coil current to increase over a long period of time. Stimulation with high frequency pulses provides a solution since it can maintain local depolarization and axonal blockage. However, delivering high frequency pulses using a large coil is challenging^49^, due to the technical difficulties encountered in delivering larger currents to such coil (much higher impedance, much higher energy-storage requirement, and much severe cooling issues). Large coils will also non-selectively stimulate many neurons and leads to unpredictable results such as inducing seizures^50^. However, with small sized coils of low induction, it is easy to deliver a large current at high frequency to the miniature coil for local stimulation. Therefore, high frequency stimulation with a miniature coil provides an interesting alternative to the conventional DC or high frequency methods with electrodes for axonal blockage.

#### Potential thermal effects of the miniature coil

The potential adverse effect of strong currents in the coil is the thermal effects and the damage of the coil and the surrounding tissue. In our experiment, the output of the power amplifier is constant voltage pulses. For a 10 V stimulus signal, the voltage drop across the coil is 2.16 V. When this constant voltage is applied to the coil, the coil current will quickly reach a steady state and the current in the coil is 1.08 A. Previous work^20^ has reported that the peak current that can damage such miniature coil is as great as 10 A, therefore there is no risk of breakdown of the coil in our experiment. The average power is 1.15 watts for the 2 Ω resistance of the coil driven by a 50% duty time square pulses.

Previous studies on invertebrate axons have found that the operating temperature in mollusks ranges from freezing temperature to 40 °C^51^, and a more than 20 °C of local temperature is needed for thermal blockage of the unmyelinated squid giant axon^52^. Heat blockage in myelinated axons happens at around 50 °C in mammalian myelinated nerves in cats^53^. In our experiments, for the 30 s experimental trial, we observe a less than 3 °C of temperature rise in a local area next to the coil. To evaluate the effect of this slight increase in temperature in axon potential propagation and axonal conductance blockage by the miniature coil, we ran our model at 23 °C and compared the results with the original model results at 20 °C (room temperature). This slight change in temperature did not affect the triggering and traveling of the action potential in the modeled axon, nor did it change the effectiveness of the coil in axonal blockage.

However, when more intense current is needed to block the axons with much smaller size (i.e., (< 2 µm, unmyelinated axons in humans)), it is essential to control the power consumption to avoid thermal effects and potential tissue damage. First, it will be ideal to choose a coil with smaller resistance and use pulses with a shorter duration to minimize the current consumption in the coil while still ensuring complete axonal blockage. Second, it is crucial to maintain good insulation of the coil, since the covered material of the coil significantly reduce the heat dissipation. Third, the integrity of the insulation around the coil also prevents the direct contact between the coil and conductive medium (*Aplysia* saline), which generates nontrivial thermal effects. We have accidentally observed that if the coil was not completely insulated, it could cause a 6–7 °C local temperature increase around the coil.

#### Impact of coil location and orientation

The induced electric field (Fig. 7b) and the activating function (Fig. 7c) generated by the coil are both dependent on the geometry and relative orientation to the axon. Therefore, the spatial resolution of local depolarization along the axon is defined by the coil size and its relative location to the axon. With improved coil design and careful positioning, it is possible to improve spatial specificity in axonal blockage with the miniature coil. For example, local bending on the coil can increase the field gradient for neural stimulation^25^.

#### Importance of coating the coil with biocompatible material

When an electrode was used for neural stimulation, an electric current flows through the inhomogeneous tissue surrounding the electrode. As demonstrated by Rattay’s pioneer work, the activating function of a point electrode depends on the conductivity changes of the medium surrounding the electrode^44^, including oxidization of the electrode, inflammation reaction of the tissue^14^, and glial scar formation^16,17^. This probably can explain the observed inconsistency in some of the DBS practices using an implanted metal electrode. In contrast, for miniature coil stimulation, the induced electric field is not dependent on the biophysical properties of the inhomogeneous tissue surrounding the coil. Coating the coil with biocompatible, soft, dielectric material will not alter the induction of the local electric field. Therefore, a miniature coil can provide much improved biocompatibility and consistency than the metal electrodes for chronic implantation.

### Future works

Further research shall address several important research questions raised by this work. First, to directly test the modeling prediction that miniature coil causes a local depolarization and blockage of action potentials, we shall use a sharp intracellular electrode to penetrate the axonal membrane and measure the local axonal membrane potential where the coil was positioned. The large sized axons of the motor neurons in *Aplysia* should provide such possibility.

Second, this work proves that the miniature coil can block large populations of axons in the same nerve, as well as axons from a single neuron. Its potential in selectively suppressing an individual and sparing others has to be investigated since differential control of different axons inside the same nerve bundle holds significant clinical potential^30^. Theoretically, transmembrane potential induced by the magnetic coil is dependent on the size of the axon^54^ or soma^47^. Larger diameter axons could be selectively affected by the magnetic coil, as transmembrane potential induced by extracellular electrode within the axon is proportional to axonal cross-section^44^. Furthermore, this paper suggests that the intensity of the induced electric field, therefore the efficacy of axonal blockage, depends on the geometrical and electrical parameters of the coil. With a better understanding of axonal neurophysiology and deliberate coil design, it could be speculated that a miniature coil could apply axonal blockage with much improved temporal and spatial precisions.

Finally, the Hodgkin/Huxley based axon model did not stimulate several ionic channel mechanisms that might cause axonal blockage. It was suggested that magnetic stimulation could lead to a profound modification in electrophysiological properties of Na^+^ channels, A-type K^+^ channels, and Ca^2+^ channels that are all vital to neural excitability^55^. High frequency stimulation could also cause excessive K^+^ release^56,57^ from the cell and depolarization blockage. More detailed ionic mechanisms can be included in the model to test if the behavior of these channels and ions will be involved in axonal blockage by the magnetic field.

Regardless of the exact mechanism, the ability to generate axonal blockage with a miniature coil may enhance clinical practice with high frequency neural stimulation. This finding raised the possibility to control motor axons by implanting the miniature coil close to the targeted axon to stop unwanted muscle contraction. It can also be used to block sensory fibers for pain relief. Axonal conduction block using HFS also has the potential for controlling abnormal propagating activity within the brain without surgical resection of white matter tracts. Understanding the mechanism of axonal blockage is essential in designing safe stimulating devices and avoid unnecessary side effects with otherwise over-dose stimulation, such as unwanted neuron firing. The miniature coil, with a cover of bio-compatible material, could provide an interesting alternative to electric stimulation with an implanted electrode.

## Methods

### Magnetic field generating system

Commercial multilayer surface mount inductors (100 nH, MLG1005SR10JTD25, TDK U.S.A. Corporation, Uniondale, NY) were selected for the study (Table 1). The two leads of the coil were soldered to two copper wires (magnetic wire 32-AWG, GC electronics, IL, L3-616). The coil was then coated with acrylatecopolymer enamel (Revlon, New York), to provide electrical insulation and water impermeability of the exposed coil terminals^58^. The coil was positioned on the tip of a glass pipette (TW150F-4, WPS), with the two copper wires running through the inside of the pipette. The pipette was then mounted on and secured to a micromanipulator for positioning the coil close to the targeted nerve. The ends of the two wires were attached to the output of a 1000 W power amplifier with a bandwidth of 70 kHz (Pyramid PB 717X 2 channel, Pyramid Car Audio, Brooklyn, NY, 11204). The amplifier was powered by a Triple Channel DC Power Supply (2231A-30-3, Keitheley). An arbitrary function generator (AFG1022, Teletronix) was used to generate a stimulation signal (Fig. 1a). The impedance of the coil was measured at the beginning and end of each experiment to test its connectivity. Potential leakage of the coating coverage was also tested by measuring the impedance of the coil to the ground.

**Table 1:** Coil parameters.

Parameter	Value
Inductance (L)	100 nH
Maximal current (I_max_)	200 mA
Resistance (R)	2 Ω
Coil length (l)	0.5 mm
Length of each rectangular loop	1 mm
Width of each rectangular loop	0.5 mm
Number of loops (N)	20

To illustrate the structure of the coil, we removed the ceramic core and epoxy coating using a published protocol^59^. Briefly, we placed the coil into a cell culture plate (24 well) and treated it with 1 mL, 40% liquid hydrofluoric acid at room temperature for 48 h. We removed the acid with a pipette and gently washed the coil three-times with deionized water. Next, we added 10 N HCL into the well for 1 h. We then removed the acid and again gently washed the coil three times with deionized water. The coil was allowed to dry in the room temperature overnight (Fig. 1b).

### In vitro electrophysiology

*Aplysia californica* (100–150 g) were obtained from Marinus Scientific (Newport Beach, CA) and were kept in the artificial seawater at room temperature (20 ± 1 °C). Animals were anesthetized by an injection of isotonic MgCl_2_ (50% of body weight). The buccal mass was dissected out in *Aplysia* saline, and the buccal ganglion was separated from the buccal mass, which all the nerves cut close to where they innervate the buccal muscles. In some experiments, the buccal ganglion was pinned caudal side up and was partially desheathed by peeling off the top layer of the sheath with fine dissection tools. In some other experiments, the buccal ganglion was completely desheathed for intracellular recording and stimulation. An artificial *Aplysia* saline solution of pH 7.4 was made in a 1 L stock with 460 mM NaCl, 55 mM MgCl_2_·H_2_O, 11 mM CaCl_2_·2H_2_O, 10 mM KCl, and 10 mM Hepes buffering agent. A dissection solution was made in a 1 L, 333 mM stock of MgCl_2_. All experiments were performed at room temperature.

Both intracellular and extracellular suction electrodes were pulled from single-barreled capillary glass using a Flaming-Brown micropipette puller (P-30, Sutter Instrument). During intracellular experiments, sharp electrodes were backed filled with 3 M potassium acetate before use. Intracellular signals were amplified using a DC-coupled amplifier (model 1600, A-M system). DC offset was eliminated and the bridge was balanced for stimulation and recording.

For extracellular recording from the soma, the size of the electrode tip was adjusted to be slightly smaller than the size of the cell bodies. Electrodes made in the same pulling protocol were also used for nerve suction recording^60^. Since the electrode tip was smaller than the diameter of the nerve, we broke this tip so that the nerve end can fit into the glass capillary. Extracellular recordings were amplified by a Model 1700 differential AC Amplifier (A-M Systems) with a gain of 1000 and filtered by a 1.0–500 Hz band pass filter. Intracellular and extracellular signals were digitized (25 kHz) by a CED 1401, recorded and analyzed by Spike 2 software (version 7.2, Cambridge Electronic Design Limited).

Action potentials in the buccal nerve II (BN2) were generated with four different protocols.

1. Stimulation of the isolated BN2 (Fig. 2a). BN2 was separated from the buccal ganglion at the point it connected to the buccal ganglion, and a suction electrode was applied to each end of the BN2. The proximal end of the axon was used for stimulation and the distal end for recording. For stimulation, 1 ms electric pulses were delivered once every 4 s. The amplitude of the stimulation was adjusted so that several large spikes were generated by each pulse, as recorded on the other end of the BN2.
2. Antidromic stimulation of the soma in the buccal ganglion (Fig. 3a). A suction electrode was applied to the distal end of BN2 for recording and stimulation. 20 Hz, 1 ms pulse trains were delivered to the BN2 for several seconds. Since the BN2 contains the axons of the majority of the motor neurons for the I1/I3/jaw muscle^32,34,40^, antidromic stimulating activated a population of soma in the ganglion.
3. Selective activation of the B6 neuron with an extracellular electrode (Fig. 4a). Located on the rostral side of the buccal ganglion (Fig. 4b), B6 is one of the largest motor neurons for the jaw closure I1/I3 muscle in *Aplysia californica* (300–400 μm in soma diameter in animals weighing 150 to 200 g). B6 neuron generated the second largest unit in the extracellular BN2 recording^32^. To activate the B6 neuron extracellularly, an extracellular electrode was applied to the ganglion sheath above the soma (Fig. 4d). To improve the spatial accessibility of the electrode to B6, the buccal ganglion was partially desheathed, by removing the top layers of the membrane. To avoid possible shifting of the electrode on the surface of the buccal ganglion, once the target neuron was identified and the electrode was positioned, a negative suction was made to anchor the electrode tip on the ganglion membrane. Electric pulses (0.1 ms in duration) were delivered to the electrode for soma stimulation. When B6 neuron was activated, both the extracellular electrode on the soma and on the axon recorded one-on-one spikes, with the soma recording leading the axon recording by a few milliseconds (Fig. 4c).
4. Activation of B6 neuron with an intracellular electrode (Fig. 5a). When B6 neurons were impaled by the intracellular electrode, a 0.5 Hz pulse train was delivered to the B6 soma to generate action potentials in the soma. The intracellular recording and the extracellular recording from BN2 demonstrated a one-on-one relationship, with the soma recording leading the axon recording by a few milliseconds (Fig. 5b).

### Magnetic stimulation of buccal nerve II with high frequency signals

The miniature coil was position next to the middle part of the BN2. The coil was positioned so that its induced electric field was in parallel to the axons, to generate effective stimulation^41,43^. 400 Hz square waves generated by an arbitrary function generator and were delivered to the power amplifier. Two stimulation intensities were tested, which were generated by 1 V (weak stimulation) or 10 V (strong stimulation) signals, respectively. The voltage across the coil was measured by a digital oscilloscope. The 10 V signal generated a 2.16 V output from the coil, and the 1 V signal generated a 0.2 V output across the coil. When square waves of different frequencies were delivered to the coil, the output voltage across the coil maintains a square wave shape when the signal frequency > 50 Hz.

To measure the local temperature in the nerve segment under coil stimulation in *Aplysia* saline, we positioned the tip of a thermocouple to the location where the coated coil contact with the BN2. The thermocouple was connected to a digital thermometer (HH11B, Omega Engineering, Norwalk, CT) to display the temperature with 0.1 °C resolution. We delivered the coil with 400 Hz stimulus for > 30 s, with the weak (1 V) and strong stimulation protocol (10 V input signal), and record the temperature difference before and after the coil was turned on.

### Electric field induced by a miniature magnetic coil

Figure 6 illustrates an unmyelinated axon under the stimulation of a miniature coil. The center of the coil is at (0,0). The coil has a radius of R. Let’s consider a point A(r, θ) on the axon, whose distance is r from the coil center O(0,0).

Magnetic field generated by the coil is calculated by Faraday’s law of induction.

1 $$\varepsilon = - \frac{{d\phi_{B} }}{dt}$$

where ε is the electromotive force (EMF) and Φ_B_ is the magnetic flux. It could also be written in an integral form (Kelvin–Stokes theorem),

2 $$\oint {\mathop{E}\limits^{\rightharpoonup} \cdot d\mathop{l}\limits^{\rightharpoonup} = - \iint {\frac{{\partial \mathop{B}\limits^{\rightharpoonup} }}{\partial t}}} \cdot d\mathop{A}\limits^{\rightharpoonup}$$

where B is the magnetic field inside the coil. E is the induced electric field, dl is an infinitesimal vector element or the path element, dA is an infinitesimal vector element of area considered. At point A, the induced electric field is in the θ direction.

3 $$E_{\theta } = \frac{{R^{2} }}{2r}\frac{\partial B}{{\partial t}}\;\left( {{\text{r }} > {\text{ R}}} \right)$$

4 $$E_{r} = 0 \left( {{\text{r}} > {\text{R}}} \right)$$

The “Ohm’s law” for an ideal inductor is

5 $$v = L\frac{dI}{{dt}}$$

where v is the instantaneous voltage across the inductor, L the inductance of the coil in Henry, and dI/dt the instantaneous current changes in A/s. For an inductor (coil) with a flowing current (I) inside, the magnetic field is calculated by

6 $$B = \mu_{0} \frac{NI}{l}$$

where N is the loop number and l the length of the coil. Combination of (5) and (6) yields

7 $$\frac{\partial B}{{\partial t}} = \frac{{v\mu_{0} N}}{Ll}$$

Combination of (3) and (7) yields

8 $$E_{\theta } = \frac{{R^{2} }}{2r}\frac{{v\mu_{0} N}}{Ll}\;r > R$$

The induced electric field can be expressed in a Cartesian basis using matrix transformation,

9 $$E(x,y) = \left[ {\begin{array}{*{20}c} {\cos \theta } & { - \sin \theta } \\ {\sin \theta } & {\cos \theta } \\ \end{array} } \right]\left[ {\begin{array}{*{20}c} {E_{r} } \\ {E_{\theta } } \\ \end{array} } \right]$$

Since $r = (x^{2} + y^{2} )^{1/2}$, we obtained

10 $$E_{x} = - \frac{{v\mu_{0} NR^{2} }}{2Ll}\frac{y}{{x^{2} + y^{2} }}$$

11 $$E_{x} = \frac{{v\mu_{0} NR^{2} }}{2Ll}\frac{x}{{x^{2} + y^{2} }}$$

The activating function, defined as the gradients of the electric field along the axon^44^, predicts the location of and speed of depolarization or hyperpolarization by the extracellular stimulation^45^. The activating function alone the axon (in the x-direction) is

12 $$AF = \frac{{\partial E_{x} }}{\partial x} = \frac{{v\mu_{0} NR^{2} }}{2Ll}x(x^{2} + y^{2} )^{ - 3/2}$$

Electric potential distribution along the axon is expressed as

13 $$V(x) = \int {E_{x} (x)dx} = \frac{{v\mu_{0} NR^{2} }}{2Ll}a\tan \left( \frac{x}{y} \right)$$

This extracellular voltage distribution will be applied to an unmyelinated axon model in NEURON simulation environment for axonal blockage.

### Multi-compartment NEURON model of an unmyelinated axon under HFS by a miniature coil

Effects of the high frequency stimulation with miniature coil were tested with a multi-compartment axon model (Fig. 6) using NEURON simulation environment package^61^. The model simulated the axon as a cylinder with 20,000 μm in length and 15 μm in diameter. The axon was divided evenly into 200 node segments (Table 2). The Hodgkin/Huxley (H/H) type of dynamics of the fast sodium, slow potassium and leakage channels in the membrane were inserted into the nodes^62^. Detailed electrical parameters (Table 3) of the modeled axon are adapted from a published model of *Aplysia* buccal neuron^31^. Action potentials were initiated in the left end of the axon (axon(0)). A recording electrode was positioned on the right end (axon(1)). For coil stimulation, the miniature coil was positioned at the middle point of the axon, 300 µm away from the axon (Fig. 6). Square waves of various frequencies and intensities were programmed and applied to the coil. The electric voltage induced by the miniature coil (Eq. (13)) was applied to the modeled axon^63^. The model was ran at room temperature (20 °C).

**Table 2:** The geometrical parameters of the NEURON model for an *Aplysia* axon.

Geometric parameter	Value
Diameter	15 µm
Number of segments	200
Length of segments	100 µm
Total axon length	200,00 µm
Location of coil center (x)	10,000 µm
Distance of coil center to axon (y)	300 µm

**Table 3:** The electric parameters of the NEURON model for an *Aplysia* axon.

Electrical parameter	Value
Membrane capacitance (*C*_m_)	1 μF/cm^2^
**Fast Na+ channels**	
Max. sodium conductance (g_Na__)	0.12 S/cm^2^
Activation term (α_m_) of m gates	− (0.1ν + 4) (exp(− 0.1ν − 4)) − 1)^−1^
Inactivation term (β_m_) of m gates	4exp (− (ν + 65)/18)
Time constant of (t_m_) m gates	3(α_m_ + β_m_) × 3^(t/10–2.0)−1^
Activation term (α_h_) of h gates	0.07exp(− 0.05ν − 3.25)
Inactivation term (β_h_) of h gates	1/(exp(− 0.1ν − 3.5) + 1)
Time constant of (t_h_) h gates	1.7 ((α_h_ + β_h_) × 3^(t/10 – 2.0))−1^
Reversal potential (*E*_Na_)	50 mV
**Slow K + channels**	
Max. conductance (g_K__)	0.036 S/cm^2^
Activation term (α_n_) of n gates	− (0.01ν + 0.55) (exp(− 0.1ν − 5.5) − 1)^−1^
Inactivation term (β_n_) of n gates	0.125exp(− (ν + 85)/80)
Time constant of (t_n_) n gates	5.6((α_n_ + β_n_)*3^(t/10 – 2.0))^−1^
Reversal potential (*E*_K_)	− 77 mV
**Leakage channels**	
Conductance (*g*_L_)	0.00028 S/cm^2^
Reversal potential (*E*_L_)	− 65 mV

*t* environmental temperature in Celsius, *v* membrane potential of a neural segment.

### Statistics

Effects of different stimulation intensities of the coil on percentage of complete blockage of BN2 activity were compared with Chi-square analysis using SigmaStat 3.01a (Systat Software, Inc.).

## Supplementary information

Supplementary Information.

Supplementary Video 1.

Supplementary Video 2.

Supplementary Video 3.

## Data Availability

All data generated or analyzed during this study are included in this published article.

## References

[1] Habbema L, Neumann HA (2008). Lidocaine: Local anaesthetic with systemic toxicity. Ned. Tijdschr. Geneeskd..

[2] Bhadra N, Kilgore KL (2005). High-frequency electrical conduction block of mammalian peripheral motor nerve. Muscle Nerve.

[3] Bhadra, N. & Kilgore, K. L. High-frequency nerve conduction block. in *Conference Proceedings: Annual International Conference of the IEEE Engineering in Medicine and Biology Society. IEEE Engineering in Medicine and Biology Society*, 4729–4732 (2004). 10.1109/IEMBS.2004.1404309. 10.1109/IEMBS.2004.1404309 17271365

[4] Kilgore KL, Bhadra N (2004). Nerve conduction block utilising high-frequency alternating current. Med. Biol. Eng. Comput..

[5] Boger A, Bhadra N, Gustafson KJ (2008). Bladder voiding by combined high frequency electrical pudendal nerve block and sacral root stimulation. Neurourol. Urodyn..

[6] Patel YA, Butera RJ (2015). Differential fiber-specific block of nerve conduction in mammalian peripheral nerves using kilohertz electrical stimulation. J. Neurophysiol..

[7] Feng Z (2017). High frequency stimulation of afferent fibers generates asynchronous firing in the downstream neurons in hippocampus through partial block of axonal conduction. Brain Res..

[8] Zheng F (2011). Axonal failure during high frequency stimulation of rat subthalamic nucleus. J. Physiol..

[9] Tandri H (2011). Reversible cardiac conduction block and defibrillation with high-frequency electric field. Sci. Transl. Med..

[10] Gaunt RA, Prochazka A (2009). Transcutaneously coupled, high-frequency electrical stimulation of the pudendal nerve blocks external urethral sphincter contractions. Neurorehabil. Neural Repair.

[11] Peckham PH, Knutson JS (2005). Functional electrical stimulation for neuromuscular applications. Annu. Rev. Biomed. Eng..

[12] Rosenbaum R (2014). Axonal and synaptic failure suppress the transfer of firing rate oscillations, synchrony and information during high frequency deep brain stimulation. Neurobiol. Dis..

[13] Jitkritsadakul O (2017). Systematic review of hardware-related complications of deep brain stimulation: Do new indications pose an increased risk?. Brain Stimul..

[14] Kim YT, Hitchcock RW, Bridge MJ, Tresco PA (2004). Chronic response of adult rat brain tissue to implants anchored to the skull. Biomaterials.

[15] Liu B (2017). Enhanced biocompatibility of neural probes by integrating microstructures and delivering anti-inflammatory agents via microfluidic channels. J. Neural Eng..

[16] Polikov VS, Tresco PA, Reichert WM (2005). Response of brain tissue to chronically implanted neural electrodes. J. Neurosci. Methods.

[17] Grill WM, Norman SE, Bellamkonda RV (2009). Implanted neural interfaces: Biochallenges and engineered solutions. Annu. Rev. Biomed. Eng..

[18] Walsh V, Pascual-Leone A (2003). Transcranial Magnetic Stimulation: A Neurochronometrics of Mind.

[19] Ye H, Steiger A (2015). Neuron matters: Electric activation of neuronal tissue is dependent on the interaction between the neuron and the electric field. J. Neuroeng. Rehabil..

[20] Bonmassar G (2012). Microscopic magnetic stimulation of neural tissue. Nat. Commun..

[21] Lee, S. W. & Fried, S. I. The response of L5 pyramidal neurons of the PFC to magnetic stimulation from a micro-coil. in *Conference Proceedings: Annual International Conference of the IEEE Engineering in Medicine and Biology Society. IEEE Engineering in Medicine and Biology Society. Annual Conference*, 6125–6128 (2014). 10.1109/EMBC.2014.6945027. 10.1109/EMBC.2014.6945027 PMC4465444 25571395

[22] Park HJ (2013). Activation of the central nervous system induced by micro-magnetic stimulation. Nat. Commun..

[23] Koivuniemi A, Wilks SJ, Woolley AJ, Otto KJ (2011). Multimodal, longitudinal assessment of intracortical microstimulation. Prog. Brain Res..

[24] Cogan SF (2008). Neural stimulation and recording electrodes. Annu. Rev. Biomed. Eng..

[25] Lee SW, Fallegger F, Casse BD, Fried SI (2016). Implantable microcoils for intracortical magnetic stimulation. Sci. Adv..

[26] Canales A (2015). Multifunctional fibers for simultaneous optical, electrical and chemical interrogation of neural circuits in vivo. Nat. Biotechnol..

[27] Saxena T (2013). The impact of chronic blood-brain barrier breach on intracortical electrode function. Biomaterials.

[28] Joseph L, Butera RJ (2009). Unmyelinated Aplysia nerves exhibit a nonmonotonic blocking response to high-frequency stimulation. IEEE Trans. Neural Syst. Rehabil. Eng..

[29] Lothet EH (2014). Alternating current and infrared produce an onset-free reversible nerve block. Neurophotonics.

[30] Lothet EH (2017). Selective inhibition of small-diameter axons using infrared light. Sci. Rep..

[31] Lu H, Chestek CA, Shaw KM, Chiel HJ (2008). Selective extracellular stimulation of individual neurons in ganglia. J. Neural Eng..

[32] Lu H, McManus JM, Chiel HJ (2013). Extracellularly identifying motor neurons for a muscle motor pool in Aplysia californica. J. Vis. Exp..

[33] Warman EN, Chiel HJ (1995). A new technique for chronic single-unit extracellular recording in freely behaving animals using pipette electrodes. J. Neurosci. Methods.

[34] Church PJ, Lloyd PE (1991). Expression of diverse neuropeptide cotransmitters by identified motor neurons in Aplysia. J. Neurosci..

[35] Church PJ, Lloyd PE (1994). Activity of multiple identified motor neurons recorded intracellularly during evoked feedinglike motor programs in Aplysia. J. Neurophysiol..

[36] Gardner D (1971). Bilateral symmetry and interneuronal organization in the buccal ganglia of Aplysia. Science.

[37] Morton DW, Chiel HJ (1993). The timing of activity in motor neurons that produce radula movements distinguishes ingestion from rejection in Aplysia. J. Comp. Physiol. A.

[38] Ye H, Morton DW, Chiel HJ (2006). Neuromechanics of coordination during swallowing in Aplysia californica. J. Neurosci..

[39] Ye H, Morton DW, Chiel HJ (2006). Neuromechanics of multifunctionality during rejection in Aplysia californica. J. Neurosci..

[40] Scott ML, Govind CK, Kirk MD (1991). Neuromuscular organization of the buccal system in *Aplysia californica*. J. Comp. Neurol..

[41] Gluckman BJ (1996). Electric field suppression of epileptiform activity in hippocampal slices. J. Neurophysiol..

[42] Chan CY, Nicholson C (1986). Modulation by applied electric fields of Purkinje and stellate cell activity in the isolated turtle cerebellum. J. Physiol..

[43] Jefferys JG (1981). Influence of electric fields on the excitability of granule cells in guinea-pig hippocampal slices. J. Physiol..

[44] Rattay F (1986). Analysis of models for external stimulation of axons. IEEE Trans. Bio-med. Eng..

[45] Lee SW, Fried SI (2017). Enhanced control of cortical pyramidal neurons with micromagnetic stimulation. IEEE Trans. Neural Syst. Rehabil. Eng..

[46] Tai C, Roppolo JR, de Groat WC (2009). Analysis of nerve conduction block induced by direct current. J. Comput. Neurosci..

[47] Ye H, Cotic M, Carlen PL (2007). Transmembrane potential induced in a spherical cell model under low-frequency magnetic stimulation. J. Neural Eng..

[48] Ackermann DM, Bhadra N, Gerges M, Thomas PJ (2011). Dynamics and sensitivity analysis of high-frequency conduction block. J. Neural Eng..

[49] Ebert U, Ziemann U (1999). Altered seizure susceptibility after high-frequency transcranial magnetic stimulation in rats. Neurosci. Lett..

[50] Rossi S, Hallett M, Rossini PM, Pascual-Leone A, The Safety of TMS Consensus Group (2009). Safety, ethical considerations, and application guidelines for the use of transcranial magnetic stimulation in clinical practice and research. Clin. Neurophysiol..

[51] Ganguly M (2019). Voltage-gated potassium channels are critical for infrared inhibition of action potentials: An experimental study. Neurophotonics.

[52] Ganguly M, Jenkins MW, Jansen ED, Chiel HJ (2019). Thermal block of action potentials is primarily due to voltage-dependent potassium currents: A modeling study. J. Neural Eng..

[53] Zhang Z (2016). Conduction block of mammalian myelinated nerve by local cooling to 15–30 °C after a brief heating. J. Neurophysiol..

[54] Ye H, Cotic M, Fehlings MG, Carlen PL (2011). Transmembrane potential generated by a magnetically induced transverse electric field in a cylindrical axonal model. Med. Biol. Eng. Comput..

[55] Tan T (2013). Repetitive transcranial magnetic stimulation increases excitability of hippocampal CA1 pyramidal neurons. Brain Res..

[56] Bikson M (2001). Suppression of epileptiform activity by high frequency sinusoidal fields in rat hippocampal slices. J. Physiol..

[57] Lian J, Bikson M, Sciortino C, Stacey WC, Durand DM (2003). Local suppression of epileptiform activity by electrical stimulation in rat hippocampus in vitro. J. Physiol..

[58] Park HJ (2013). Activation of the central nervous system induced by micro-magnetic stimulation. Nat. Commun..

[59] Ye H, Chen VC, Helon J, Apostolopoulos N (2020). Focal suppression of epileptiform activity in the hippocampus by a high-frequency magnetic field. Neuroscience.

[60] Johnson BR, Hauptman SA, Bonow RH (2007). Construction of a simple suction electrode for extracellular recording and stimulation. J. Undergrad. Neurosci. Educ..

[61] Hines ML, Carnevale NT (1997). The NEURON simulation environment. Neural Comput..

[62] Hodgkin AL, Huxley AF (1952). A quantitative description of membrane current and its application to conduction and excitation in nerve. J. Physiol..

[63] Joucla S, Gliere A, Yvert B (2014). Current approaches to model extracellular electrical neural microstimulation. Front. Comput. Neurosci..

