# Supplementary material for: Axonal blockage with microscopic magnetic stimulation
Source: Sci Rep. 2020 Oct 22;10:18030. doi: 10.1038/s41598-020-74891-3 (PMC7582966; doi:10.1038/s41598-020-74891-3)
Supplement: Supplementary file 1 — Supplementary Information. [file 41598_2020_74891_MOESM1_ESM.docx]

**Axonal blockage with microscopic magnetic stimulation**

Jorden Skach, Catherine Conway, Lauryn Barrett and Hui Ye*

Department of Biology, Loyola University Chicago, Chicago, IL. USA

**Supplementary Information:**

Supplementary video 1: NEURON modeling video. An action potential propagates along an unmyelinated Aplysia motor axon.

Supplementary video 2: NEURON modeling video. 400 Hz, sub-threshold stimulation of an unmyelinated axon with a miniature coil. Coil stimulation cannot block action potential traveling in the axon.

Supplementary video 3: NEURON modeling video. 400 Hz, supra-threshold stimulation of an unmyelinated axon with a miniature coil. Coil stimulation blocks action potential traveling in the axon.
